# High Oxygen Level in a Soaking Treatment Improves Early Root and Shoot Development of Black Willow Cuttings

**DOI:** 10.1100/tsw.2004.144

**Published:** 2004-10-22

**Authors:** L.T. Martin, S.R. Pezeshki, F.D. Shields

**Affiliations:** ^1^ Department of Biology, The University of Memphis, Memphis, TN 38152, USA; ^2^ National Sedimentation Laboratory, USDA-ARS, Oxford, MS 38655, USA

**Keywords:** adventitious root formation, black willow, cuttings, riparian restoration, Salix nigra

## Abstract

Black willow (Salix nigra) stem cuttings are commonly used to stabilize eroded streambanks with survival dependent on rapid development of adventitious roots to maintain plant water balance, absorb nutrients, and provide anchorage and support especially during flood and drought events. Soaking cuttings in water prior to planting increases survival and growth rates, but it is not known whether oxygen content in the soaking water affects the rate of early root and shoot initiation and growth. A laboratory experiment tested the hypothesis that cuttings treated with high oxygen (>95% saturation, 8.62 mg O_2_ l—^-1^) soaking exhibit more rapid initiation and growth of roots and shoots than cuttings treated with low oxygen (<15% saturation, 1.24 mg O_2_ l^-1^) soaking and control (unsoaked). Root initiation was enhanced in both high and low O_2_ soaking treatments compared to control (100, 93, and 41%, respectively, n = 27). High O_2_ soaking led to greater root length than low O_2_ soaking during the fourth week after planting (26.5 and 12.3 cm on day 22; 27.7 and 19.1 cm on day 27, respectively). Shoot growth was greater in high O_2_ compared to low O_2_ soaking on days 36 and 56 after planting (9.3 and 6.3 cm on day 36, 10.7 and 7.2 cm on day 56, respectively). Shoot and root biomass production was stimulated in both soaking treatments, with 200% more biomass production by day 59 compared to control. Results of this study demonstrated that a high oxygen soaking treatment has potential for improving early root and shoot growth, and survival in willow cuttings planted at riparian restoration sites.

## INTRODUCTION

Restoration of eroded streambanks using black willow (*Salix nigra* Marshall) stem cuttings is inexpensive ($30 $m^{-1}$) compared to traditional channel stabilization methods costing up to $300 $m^{-1}$[1,2,3]. However, successful restoration using stem cuttings is dependent on a well-developed root system to survive flooding and drought stresses that are common in these dynamic ecosystems[4,5,6,7,8,9,10,11,12]. Root anchorage and support are also critical to survival of stem cuttings in active floodplains. Correlations were found between flooding tolerance and uprooting resistance in willow and cottonwood species, and distribution patterns on floodplains[13,14].

Soaking promotes rapid development of roots and shoots[15,16,17]. Black willow cuttings soaked in water for 10 days had significantly greater shoot, leaf, root, total biomass, and survival rates than cuttings soaked for 0 or 3 days[18]. In a field study, cuttings soaked for 14 days in a nearby pond had a higher rate of survival than unsoaked cuttings at the end of the first growing season (64 and 53%, respectively)[19].

Willows develop adventitious roots from preformed primordia in nodal regions with plant growth regulators, ethylene and auxin, playing important roles in the initiation and development process[20,21,22,23]. For example, biosynthesis of ethylene is stimulated by auxins and environmental stresses, including flooding. In flooded conditions, ethylene accumulates in plant tissues due to restricted diffusion rates in water, stimulating or inhibiting adventitious root formation (ARF) depending on concentration[11,24,25]. Soaking dormant, woody stem cuttings in water may initiate a chain of metabolic processes similar to flooding, resulting in production of ethylene and stimulation of ARF. Greater ARF was reported in swamp tupelo (*Nyssa sylvatica* var. *biflora* [Walt.] Sarg.) seedlings in flowing water compared to stagnant water conditions[26]. Flowing water is likely to have higher oxygen content than stagnant water and may have contributed to greater ARF.

It is not known whether oxygen level in a soaking treatment affects rate of early root and shoot development in black willow cuttings. If a high oxygen soaking treatment improves rates of root and shoot initiation and development in cuttings, this information could be useful in improving survival rates at restoration sites. For example, such improvements may be achieved by soaking cuttings in a flowing stream with high oxygen content instead of a stagnant pond with low oxygen content. The purpose of this experiment was to test the hypothesis that a high oxygen soaking treatment (>95% $O_2$ saturation) stimulates early root and shoot initiation, growth, and survival of black willow cuttings compared to a low oxygen soaking treatment (<15% $O_2$ saturation) or control (unsoaked).

## MATERIALS AND METHODS

Dormant black willow cuttings with a basal diameter of 1.2–1.5 cm were collected from the floodplain of Big Creek, a tributary to the Loosahatchie River in Millington, TN on September 26, 2002 and trimmed to a length of 20 cm. Multiple cuttings were collected from each donor tree to ensure that each genotype was represented in all treatment groups. Secondary branches were removed. Cuttings were divided into 3 treatment groups with 48 replicates in each group: (1) control group with no soaking treatment, (2) a low $O_2$ soaking treatment, and (3) a high $O_2$ soaking treatment. Cuttings in the control group were kept moist and cool, while treated cuttings were bundled and soaked under water for 10 days. Low $O_2$ cuttings were soaked using nitrogen gas bubbled into the water to reduce dissolved oxygen content to <15% oxygen saturation ($1.24 \pm 0.40$ mg $O_2$ $l^{-1}$). High $O_2$ cuttings were soaked using air bubbled into the water to increase dissolved oxygen content to >95% oxygen saturation ($8.62 \pm 0.20$ mg $O_2$ $l^{-1}$). Dissolved oxygen content was measured daily using a YSI model 95, dissolved oxygen meter (Yellow Springs Instruments, Yellow Springs, OH). On day 6 of the soaking treatment, cuttings were rinsed with water to remove any accumulation of bacteria or fungi and placed in treatment tanks with clean water.

Following treatment, cuttings were planted one per pot and arranged in a completely randomized design in the laboratory. Pots were 10 cm in diameter and 40 cm in height, and filled with washed sand. Liquid fertilizer (Peter's Professional) was applied to plants every 2 weeks (20 ml) at a concentration of 2.5 g/gal of water. The three treatment groups were maintained in well-watered and drained conditions. Dual-range fluorescent and incandescent lights provided an ambient light level of 200 μmol $m^{-2}$ $s^{-1}$ during a 12-h photoperiod at ambient temperature of $20 \pm 2^{o}$C.

Early root and shoot development was quantified with the following measurements. Number of shoots and tallest shoot growth were determined on days 14, 22, 27, 36, and 56 (n = 30). Tallest shoot growth was calculated by subtracting cutting height from total shoot height. Root and shoot biomass and length of longest root were determined by destructive subsampling of randomly selected plants, on days 16, 22, 27 (six replicates on each day), and day 59 (nine replicates) after planting.

Physiological measurements were conducted using a portable gas exchange analyzer (CIRAS 1, PP Systems, Hertfordshire, U.K.) to measure rates of net photosynthesis ($P_N$, μmol $CO_2$ $m^{-2}$ $s^{-1}$) and stomatal conductance ($g_s$, mmol $H_2O$ $m^{-2}$ $s^{-1}$) on days 46 and 59 after planting (eight replicates on each day). Leaf chlorophyll content (LCC) was also measured on days 46 and 59 after planting using a chlorophyll content meter (Opti-Sciences CCM-200, Tyngsboro, MA, eight replicates on each day).

Multivariate repeated measures analysis was used to determine the effects of soaking treatment and sampling date on growth of tallest shoot and number of shoots[27,28]. MANOVA was used to determine the effects of soaking treatment and sampling date on root biomass, shoot biomass, and length of longest root[28]. One-way ANOVA was used to analyze the effect of soaking treatment on $P_N$, $g_s$, and LCC. Tukey's posthoc procedure provided pairwise comparisons between treatment groups from significant ANOVAs ($p < 0.05$).

Net carbon fixation per plant was determined for each treatment group on day 59 (n = 8). Mean shoot biomass (g) was multiplied by specific leaf area ($cm^2$ $g^{-1}$) to determine leaf surface area ($cm^2$)[9]. The units for leaf surface area were converted to $m^2$ and multiplied by mean carbon fixation rate (μmol $CO_2$ $m^{-2}$ $s^{-1}$) for each treatment group to determine carbon fixation rate per plant (μmol $CO_2$ $s^{-1}$). One-way ANOVA was used to test for significant differences in carbon fixation rate per plant among the three treatment groups. Statistical procedures were performed using SPSS statistical software (version 11.5 for Windows)[27,29].

## RESULTS

MANOVA analysis found soaking treatment had a significant effect on early development of length of longest root, root biomass, and shoot biomass ($p < 0.001$, $p = 0.008$, $p = 0.003$, Wilks, $p < 0.001$) across sampling days 16, 22, and 27 after planting. Longest root length was greater in high $O_2$ than in low $O_2$ treatments on days 22 and 27 (Fig. 1). Enhanced root growth was found in both soaking treatments compared to control on day 59 ($p = 0.011$, Fig. 1).

Root biomass was stimulated in soaked treatments compared to control (Fig. 2). This trend strengthened by day 59 with 200% more root biomass produced by soaked cuttings compared to control ($p = 0.006$). Shoot biomass followed a similar trend with an early advantage in soaked treatments compared to control. Soaked treatments produced 200% more shoot biomass than control by day 59 ($p = 0.004$, Fig. 3). Higher root and shoot biomass was found in low $O_2$ cuttings compared to high $O_2$ cuttings on day 16. This was an unexpected finding that was only observed on one sampling date. It is possible that the random sampling procedure resulted in selection of six above-average cuttings from the low $O_2$ group and six below-average cuttings from the high $O_2$ group on this date.

A significant interaction was found between soaking treatment and sampling date in a repeated measures analysis of the effects of these two variables on number of shoots and tallest shoot growth (Wilks, $p = 0.003$). Shoot growth contributed to the significant interaction ($p < 0.001$), but number of shoots did not ($p = 0.133$). Significant differences in shoot growth were found among treatment groups (soaking) on all five sampling dates (day 14, $p = 0.007$; day 22, $p < 0.001$; day 27, $p < 0.001$; day 36, $p < 0.001$; and day 56, $p < 0.001$). On days 22 and 27 after planting, lower shoot growth was found in the control (no soaking) treatment compared to both high and low $O_2$ soaking treatments (Fig. 4). Differences in shoot growth became more pronounced by days 36 and 56 with greatest shoot heights in the high $O_2$ treatment (Fig. 4). Shoot heights in the low $O_2$ treatment were less than the high $O_2$ treatment, and greater than the control on days 36 and 56 (Fig. 4).

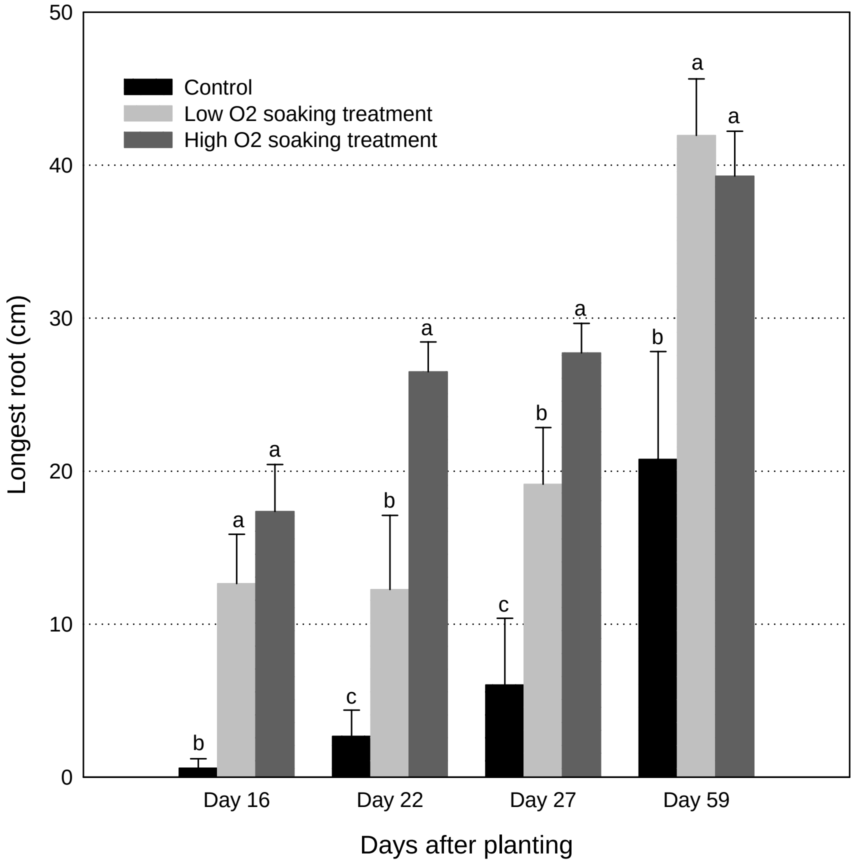

**FIGURE 1.** Mean length of longest root (cm, ± s.e.) of black willow (*S. nigra*) cuttings in control, low $O_2$ and high $O_2$ soaking treatments for combined subsampling data on days 16, 22, 27, and 59 after planting (n = 27). Different lower case letters above each bar represent significant differences between treatment groups ($p \leq 0.05$).

No differences were found in $P_N$, $g_s$, or LCC among treatment groups for days 46 and 59 combined ($p = 0.256$, $p = 0.190$, and $p = 0.952$, respectively, Table 1). However, only cuttings with healthy, well-developed leaves were selected for these measurements. Even though $P_N$ was similar among treatment groups, net carbon fixation rate per plant (µmol $CO_2$ $s^{-1}$) in both soaked treatments was double the rate of carbon fixation in unsoaked cuttings on day 59 (0.049 ± 0.008 in high $O_2$ group, 0.045 ± 0.004 in low $O_2$, and 0.022 ± 0.009 in control, $p = 0.043$). This finding explains differences in biomass accumulation between soaked and unsoaked treatments.

Soaked cuttings had high rates of root initiation compared to control (100% for high $O_2$ group, 93% for low $O_2$ group, and 41% for control, across biomass subsampling days 16, 22, 27, and 59, n = 27, Table 2).

## DISCUSSION

Soaking stimulated production of root and shoot biomass, root growth, and shoot height in this study. Increased shoot growth in the high $O_2$ group became more evident with the progression of the experiment on days 36 and 56. Shoot height is important in the acquisition of radiant energy for photosynthesis. Productivity in two *Populus* spp. was influenced by the production and retention of leaf area[30]. Although $P_N$, $g_s$, and LCC were similar in the three treatment groups in the present study, carbon fixation

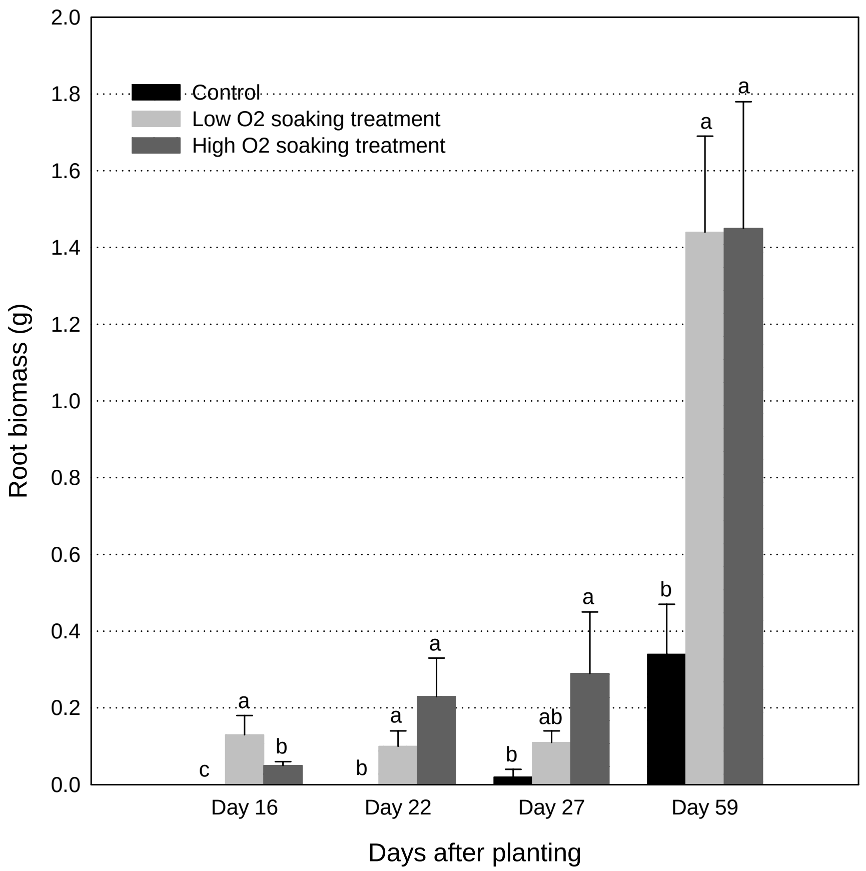

**FIGURE 2.** Mean root biomass (g, ± s.e.) of black willow (*S. nigra*) cuttings in control, low $O_2$ and high $O_2$ soaking treatment groups for combined subsampling data on days 16, 22, 27, and 59 after planting (n = 27). Different lower case letters above each bar represent significant differences between treatment groups ($p \leq 0.05$).

per plant on day 59 was clearly stimulated by both soaking treatments. Net carbon fixation per plant was enhanced 222 and 204% in high and low $O_2$ treatments compared to control. Early root and shoot growth was more important in growth potential than photosynthetic capacity or, in agreement with data previously reported for other woody species[31].

Soaking treatments produced longer root lengths compared to control during this study, with an advantage in the high $O_2$ treatment on days 22 and 27 compared to the low $O_2$ treatment. By day 59, no difference in root length was detectable between the high and low $O_2$ soaking treatments; however, both soaking treatments produced greater root lengths than the control. It is possible that root length in soaked treatments was limited by the size of the pot by day 59. Root development is important in maintaining plant water balance and in supplying nutrients to developing tissues[23]. Root length is an important factor in the plant's ability to explore soil and absorb minerals and water, especially in nutrient-poor soils and during periodic droughts that are typical in riparian habitats[25]. The early advantage of both soaking treatments in producing longer roots may aid cutting survival in an environment where flooding and drought stresses are common and cuttings are vulnerable during early root development.

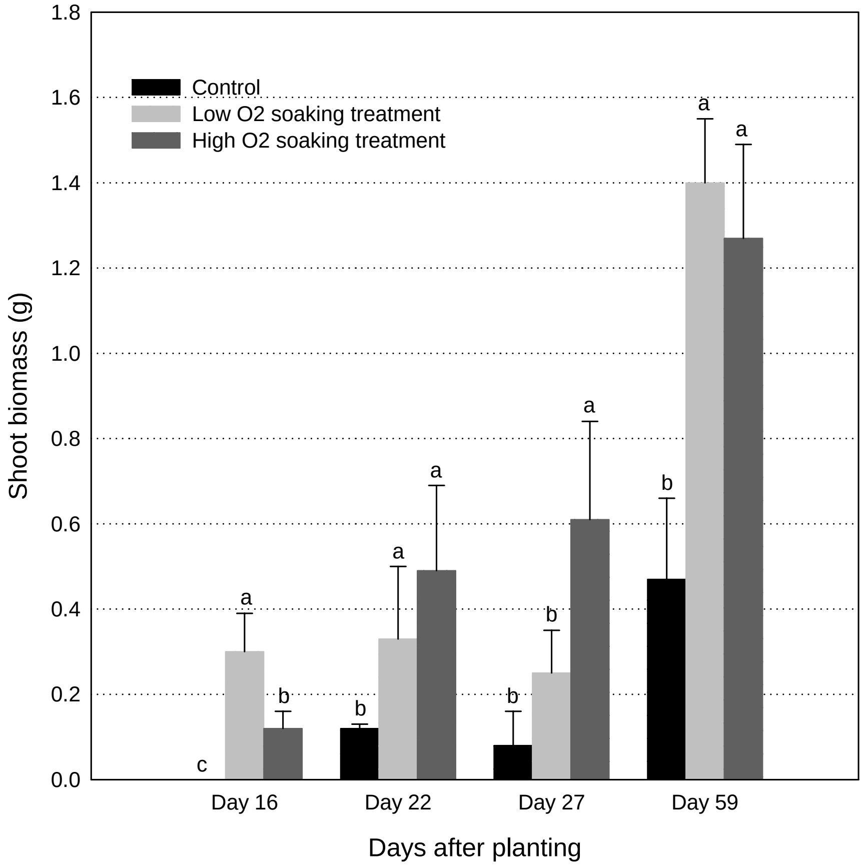

**FIGURE 3**. Mean shoot biomass (g, ± s.e.) of black willow (*S. nigra*) cuttings in control, low $O_2$ and high $O_2$ soaking treatment groups for combined subsampling data on days 16, 22, 27, and 59 after planting (n = 27). Different lower case letters above each bar represent significant differences between treatment groups ($p \leq 0.05$).

Rapid, early root development has been positively correlated with later plant productivity in woody species[16,32]. Root initiation was enhanced by soaking treatment in this experiment in both high and low $O_2$ treatment groups compared to control (100, 93, and 41%, respectively, n = 27). It has been hypothesized that ARF is triggered by increased ethylene level in the shoot resulting from a root to shoot signal, possibly 1-aminocyclopropane-1-carboxylic acid (ACC)[33,34,35,36]. The synthesis of ACC in the roots is enhanced under anoxic conditions. However, conversion of ACC to ethylene requires $O_2$ and ethylene synthesis is discontinued when $O_2$ is no longer available[37]. Greater initiation of mesocotyl roots in *Echinochloa phyllopogon* was stimulated by hypoxic conditions during flooding compared to anoxic and ethylene treatments, and aerated control[38].

Results from this experiment provided detailed, quantitative data supporting the hypothesis that soaking willow cuttings in highly oxygenated water promotes more rapid early root and shoot development compared to soaking in water with low oxygen content. In addition, both soaking treatments outperformed the control (no soaking group) supporting the results of previous studies that soaking improves early survival and growth of dormant, woody cuttings[15,18].

One practical application of our findings, for example, is that a project manager at a restoration site should use fast-flowing, highly-oxygenated, stream water for preplanting soaking of willow cuttings rather than stagnant pond water with low oxygen content to improve the rate of early root and shoot development. Clearly, rapid establishment is critical for willow cuttings to survive in eroded riparian habitats characterized by frequent drought and flooding events that impose substantial stress on plants.

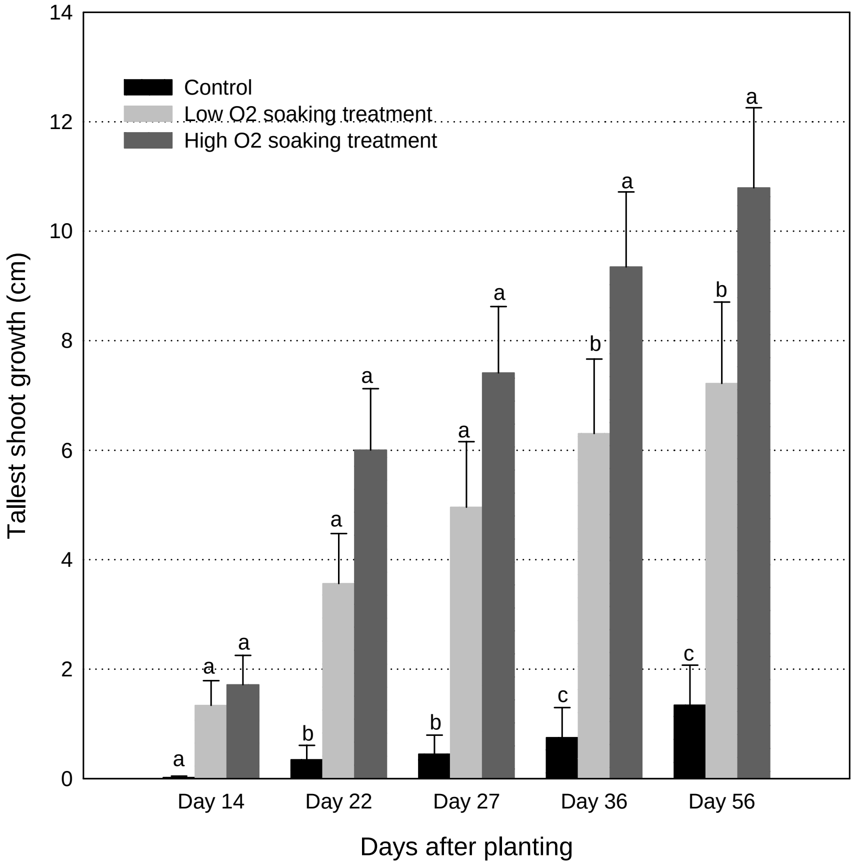

**FIGURE 4**. Mean growth of tallest shoot (cm, ± s.e.) of black willow (*S. nigra*) cuttings in control, low $O_2$ and high $O_2$ soaking treatment groups for days 14, 22, 27, 36, and 56 after planting (n = 30). Different lower case letters above each bar represent significant differences between treatment groups on each day ($p \leq 0.05$).

**TABLE 1**
**Mean (± s.e.) Net Photosynthesis ($P_N$, $\mu$mol $CO_2$ $m^{-2}$ $s^{-1}$, n = 18), Stomatal Conductance ($g_s$, mmol $H_2O$ $m^{-2}$ $s^{-1}$, n = 18), and LCC (chlorophyll content index units, n = 38) of Black Willow (*S. nigra*) Cuttings in Control, Low $O_2$ and High $O_2$ Soaking Treatment Groups on Days 46 and 59 (Combined) After Planting**

| Treatment | $P_N$ | $g_s$ | LCC |
|---|---|---|---|
| Control | 7.28 ± 0.77 | 102.33 ± 12.38 | 6.70 ± 0.36 |
| Low $O_2$ soaking | 5.26 ± 0.69 | 66.26 ± 14.15 | 6.58 ± 0.38 |
| High $O_2$ soaking | 6.26 ± 0.97 | 82.55 ± 14.14 | 6.76 ± 0.46 |

No significant differences were found among treatment groups using one-way ANOVA to test for the effect of soaking treatment on measured variables ($p$ = 0.256 for $P_N$, $p$ = 0.190 for $g_s$, and $p$ = 0.952 for LCC).

**TABLE 2**
**Percent of Black Willow (*S. nigra*) Cuttings with Observed ARF During Random Biomass Subsampling on Days 16, 22, and 27 (n = 6), and day 59 (n = 9)**

| | Biomass Subsampling | | | |
|---|---|---|---|---|
| **Treatment** | **Day 16** | **Day 22** | **Day 27** | **Day 59** |
| Control | 17% | 50% | 33% | 56% |
| Low $O_2$ soaking | 100% | 67% | 100% | 100% |
| High $O_2$ soaking | 100% | 100% | 100% | 100% |

## ACKNOWLEDGMENTS

This work was supported in part by a grant from the USDA-ARS National Sedimentation Laboratory in Oxford, MS, Contract Number 58-6408-1-098. Partial support was also provided by a grant from The University of Memphis, Faculty Research Fund. This support does not necessarily imply endorsement by the University of research conclusions. The authors appreciate the assistance of students in collecting data for this project, particularly Scott Munsell and Maia Ferguson. Assistance with statistical analyses provided by Dr. Scott Franklin and Dr. Maciej Biernacki is also appreciated.
